# Boiling Heat Transfer Evaluation in Nanoporous Surface Coatings

**DOI:** 10.3390/nano11123383

**Published:** 2021-12-13

**Authors:** Uzair Sajjad, Imtiyaz Hussain, Muhammad Imran, Muhammad Sultan, Chi-Chuan Wang, Abdullah Saad Alsubaie, Khaled H. Mahmoud

**Affiliations:** 1 Department of Mechanical Engineering, National Yang Ming Chiao Tung University, 1001 University Road, Hsinchu 300, Taiwan; ccwang@nctu.edu.tw; 2 Department of Power Mechanical Engineering, National Tsing Hua University, No. 101, Section 2, Guangfu Road, East District, Hsinchu City 300, Taiwan; imtiyazkou@yahoo.com; 3 Department of Mechanical, Biomedical and Design Engineering, Aston University, Birmingham B4 7ET, UK; 4 Department of Agricultural Engineering, Bahauddin Zakariya University, Bosan Road, Multan 60800, Pakistan; muhammadsultan@bzu.edu.pk; 5 Department of Physics, College of Khurma University College, Taif University, P.O. Box 11099, Taif 21944, Saudi Arabia; asubaie@tu.edu.sa (A.S.A.); k.hussein@tu.edu.sa (K.H.M.)

**Keywords:** boiling heat transfer, nanoporous coating, deep learning

## Abstract

The present study develops a deep learning method for predicting the boiling heat transfer coefficient (HTC) of nanoporous coated surfaces. Nanoporous coated surfaces have been used extensively over the years to improve the performance of the boiling process. Despite the large amount of experimental data on pool boiling of coated nanoporous surfaces, precise mathematical-empirical approaches have not been developed to estimate the HTC. The proposed method is able to cope with the complex nature of the boiling of nanoporous surfaces with different working fluids with completely different thermophysical properties. The proposed deep learning method is applicable to a wide variety of substrates and coating materials manufactured by various manufacturing processes. The analysis of the correlation matrix confirms that the pore diameter, the thermal conductivity of the substrate, the heat flow, and the thermophysical properties of the working fluids are the most important independent variable parameters estimation under consideration. Several deep neural networks are designed and evaluated to find the optimized model with respect to its prediction accuracy using experimental data (1042 points). The best model could assess the HTC with an R^2^ = 0.998 and (mean absolute error) MAE% = 1.94.

## 1. Introduction

Pool boiling is an efficient thermal management process for high-flux applications such as power plants, desalination, electronics cooling, and the like. The performance of nucleate boiling is strongly related to the surface morphology of the boiling surface, working fluids, operating conditions, and supplied heat flux. For enhancement of the boiling heat transfer, either active or passive techniques have been employed. Yet, passive techniques via surface engineering, in particular, have attracted the most attention for their cost-effectiveness. In practice, boiling surfaces can be made available through additive, subtractive, or compound manufacturing methods. The main purpose of the structured surfaces is to increase nucleation sites’ density as much as possible. One of the most commonly used surfaces adopts controllable coating surfaces. Surface morphology contains microscale, nanoscale, or other features reported in the literature [1]. Micro- and nanoscale surfaces are produced using numerous fabrication techniques, including electron beam deposition [2], physical vapor deposition [3], sandblasting [4], polishing and machining [5], sintering [6], compound fabrication [7], and other surface engineering techniques [1]. These engineered surfaces offer boiling augmentation owing to their different enhancement mechanisms [8]. The nanoporous surface, in particular, has drawn the attention of researchers recently due to its significant heat transfer augmentation. A large number of nanoporous coating materials produced from several fabrication methods on a variety of substrates have been proposed for boiling enhancement. Various nanoporous coated surfaces for a range of morphological parameters have exhibited a significant improvement in the enhancement of the heat transfer coefficient (HTC) and critical heat flux (CHF), and a reduction in the onset of nucleate boiling (ONB). A group of researchers reported a substantial enhancement in HTC and CHF for an anodized Al_2_O_3_ or alumina nanoporous coating on an aluminum substrate [9]. They reported that the liquid spreading and high capillary pressure due to the porous wicks were responsible for the improved CHF. In their studies, the authors evaluated the hydrophobic self-assembly mono-layered alumina nanoporous surfaces [10], sponge-like nano-porous structures [11], and three dimensionally-interconnected alumina nanoporous surfaces [12], reporting a substantial enhancement in HTC.

Vemuri and Kim [13] tested an alumina-coated nanoporous aluminum surface with an FC-72 dielectric liquid to yield up to a 30% reduction in ONB. Rahman et al. [14] used biological templates to fabricate nanoporous nickel structures on various substrates such as gold, aluminum, copper, and stainless steel. They achieved up to 200% enhancement in CHF and HTC for water without any performance degradation for 24 h boiling. By using the hot-dip galvanizing and de-alloying method, nanoporous copper surfaces with a pore diameter of 50–200 nm were created to reduce up to 63% incipience superheat and 172% enhancement in HTC [15]. Annealing and electrodeposition were used to fabricate nanoporous copper dendrites of increased strength for enhanced boiling of water [16]. The flower-like ZnO structures were coated by the MAND (microreactor-assisted nanomaterial deposition) technique on copper and aluminum substrates, which yielded a four-fold improvement in CHF and a noticeable reduction in wall superheat [17]. In another study, electroplating was employed to produce Cu_2_O (fractal-like) structures on a NiCr surface with controlled wettability. These surfaces demonstrated improved boiling performance with water [18]. Some of the other employed methods for fabrication of the enhanced nanoporous surfaces were anodization and pyrolysis [14], cold spraying [19], electrostatic spraying and sintering [17], chemical vapor deposition [18], and anodizing [20]. The literature review suggests that nanoporous surfaces possess a great boiling enhancement potential for offering tremendous nucleation site density, as well as wettability and bubble dynamics control. In addition, improvements in HTC, CHF and ONB can be achieved by adequately engineering the nanoporous surfaces for different working fluids. Nanoporous coated surfaces offer multi-dimensional enhancements in CHF and HTC and eliminate temperature excursion. Hence, they are considered to be a solution for the tailoring of high-flux applications. It is therefore crucial to evaluate the boiling performance of various nanoporous surfaces produced by different fabrication methods. However, some efforts have been made recently for the prediction of the critical heat flux or heat transfer coefficient by artificial intelligence techniques for nanofluids [21], sintered coated microporous surfaces [22], nanorefrigerants [23], and so on.

The literature review suggests that there are no empirical–mathematical models available in the literature that can accurately predict the pool boiling performance of various kinds of coated nanoporous surfaces. Therefore, the objective of this study is to use an artificial intelligence technique (e.g., deep learning) for the accurate prediction of the HTC of nanoporous surfaces for a variety of substrates and coating materials subject to saturated pool boiling conditions. The prediction is extended to a wide range of the investigated parameters.

## 2. Materials and Methods

In this section, information regarding data collection, classification, evaluation of the data through feature engineering, and finding of the optimized model for predicting the heat transfer coefficient of the nanoporous coated surfaces (training, testing, and evaluation) has been provided. After finding the optimal network, the unseen data are used to validate the accuracy of the developed model.

### 2.1. Data Collection

In this study, the authors collected test results from several sources in the literature [14,16,18,19,20,21,23,24,25,26,27,28,29,30,31,32,33,34,35,36,37,38,39]. These works report the pool boiling performance of different working fluids such as water, dielectric liquids, and liquid nitrogen subject to different types of nanoporous coated surfaces. These surfaces were fabricated on a variety of substrates (tubular and plain), such as copper, aluminum, gold, teflon, stainless steel, and zirconium, etc. Several fabrication methods, such as electro polishing and anodizing, hot-dip galvanizing and de-alloying (HDGD), using biological templates, chemical vapor deposition (CVD), alloying, and de-alloying, etc., were used to produce these coatings. A database including 1042 experimental data points was used for the assessment of the heat transfer coefficient (HTC) of the nanoporous coated surfaces subjected to different working fluids. The range of all the investigated parameters (both input and output) is given in Table 1. It can be seen that the investigation has been extended to a wide range of heat fluxes (0.285–2095 kW/m^2^), substrate materials (thermal conductivity ranges from 0.25 to 401 W/m·K), and pore diameters (10–200 nm).

### 2.2. Feature Engineering and Data Visualisation

Some of the important morphological parameters of the surface were taken into account. Feature engineering identification was then performed to reduce the number of features. After features engineering, only the most influential parameters were considered for the modelling of the HTC. These input features included the surface properties (such as pore diameter and thermal conductivity of the substrate), liquid properties (such as boiling point, the heat of vaporization, surface tension, specific heat, liquid thermal conductivity, and liquid density), and pool boiling conditions (saturation at atmospheric pressure, wall superheat, and heat flux). By setting the surface properties, liquid properties, and testing conditions as the input parameters, the HTC of different kinds of nanoporous coated surfaces subject to various working fluids can be predicted accurately by using deep learning (DL). The unavailability and diversity of mathematical–empirical models in the literature suggest that an artificial intelligence (AI)-based model is more appropriate to predict the BHTC (boiling heat transfer coefficient) with complex interactions amid various nanoporous coated surfaces, operating conditions, and work fluids. A deep learning model was therefore developed (trained, tested, and evaluated). The proposed method is able to predict the variety of coated porous nanostructures with great accuracy.

**Figure 1:**
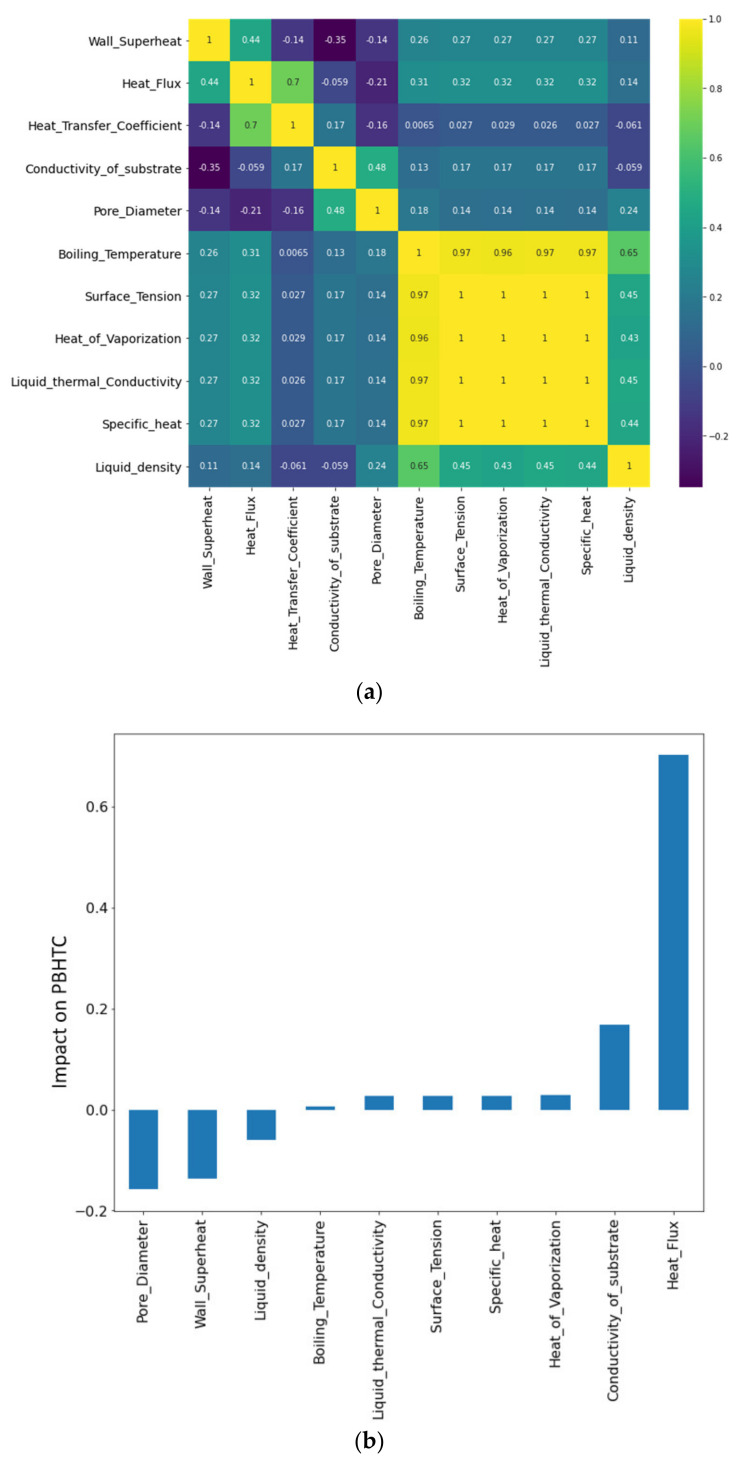
Features correlation (**a**) heat map and (**b**) correlation chart.

Input and output parameters can be correlated by a heat map and correlation chart, as shown in Figure 1. The correlation chart is plotted using the Pearson parametric correlation [9]. The heat flux and conductivity of the substrate are positively correlated with the HTC, but the pore diameter and wall superheat are negatively correlated with the HTC.

**Figure 2:**
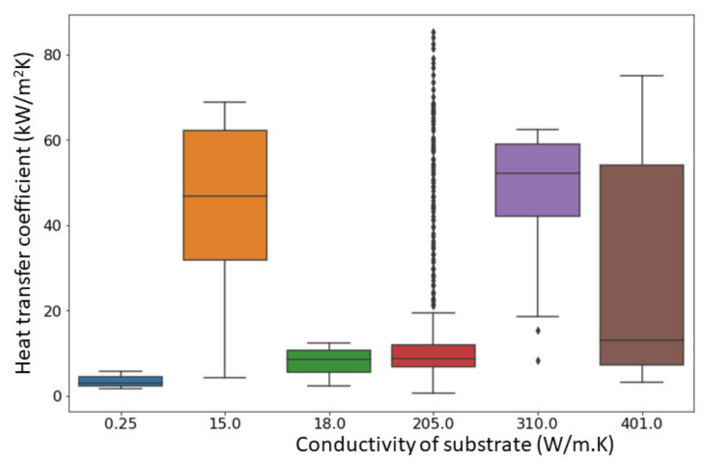
Heat transfer coefficient vs. thermal conductivity of substrate material.

Figure 2 illustrates the reported heat transfer coefficient vs. the substrate material’s thermal conductivity for nanoporous surfaces fabricated by different methods. The trend of the experimental results clearly shows that relatively higher HTCs were achieved for the nanostructures fabricated on the substrates with high thermal conductivities.

**Figure 3:**
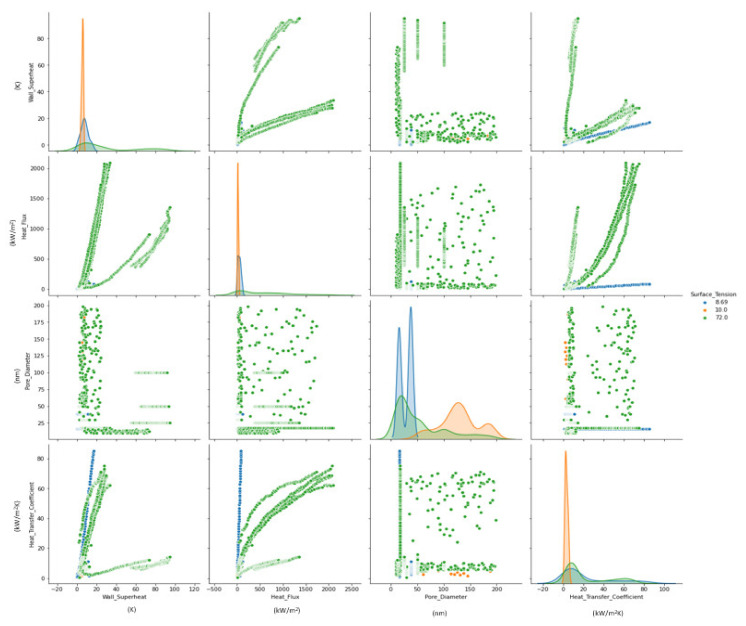
Experimental data distribution of various parameters.

Figure 3 illustrates the experimental data distribution of various parameters such as wall superheat, heat flux, pore diameter, and heat transfer coefficient for three investigated working fluids (dielectric liquids, DI water, and liquid nitrogen). Figure 3 clearly shows the considerable dispersion of the experimental data with related parameters, suggesting the infeasibility of simple correlations to describe such diversities.

### 2.3. Developing and Training the DL Model

**Figure 4:**
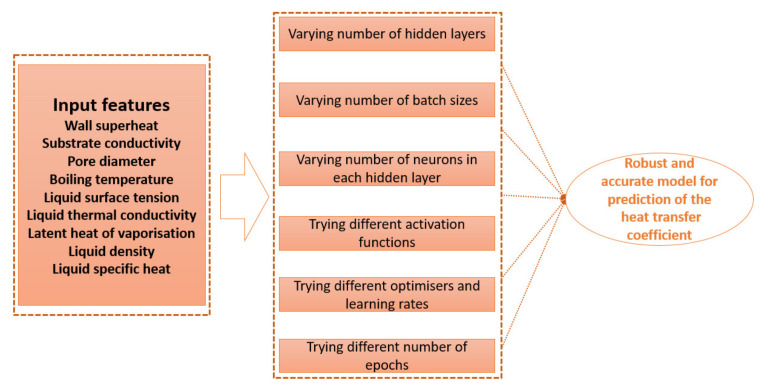
Procedure for finding the optimal model for estimation of HTC for nanoporous surfaces.

The details regarding the training process of the DL method are available in our previous study [22]. The experimental data were divided into 80/20 for the training and testing. The steps and procedure to find the optimal model for the HTC prediction is shown in Figure 4.

**Figure 5:**
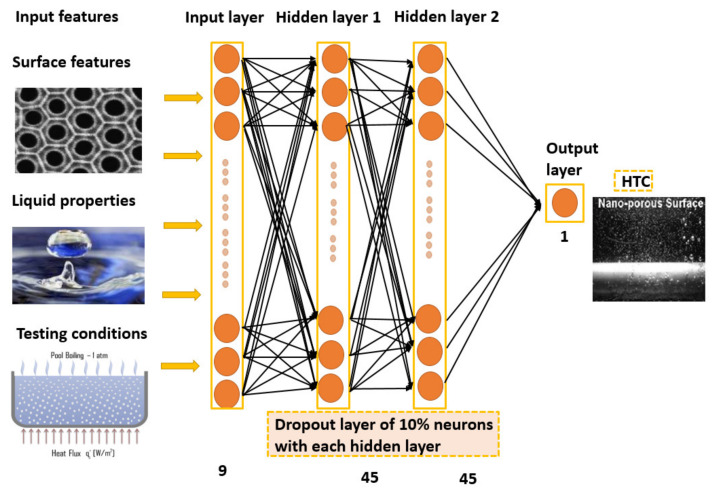
The optimal structure.

**Figure 6:**
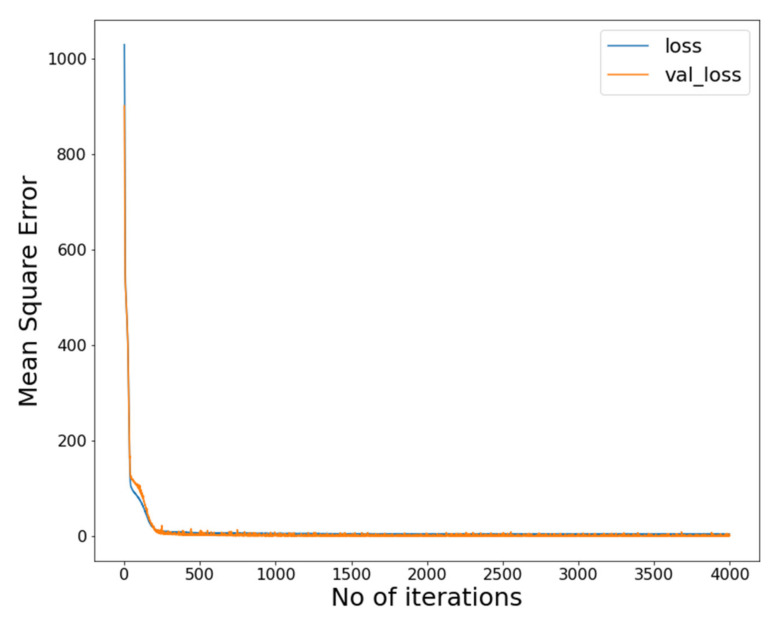
MSE trend.

The different activation functions were tried and different learning rates were investigated. The final model had an architecture of 1-2-1 with (45–45 dense neurons). The overfitting caused by a large number of neurons was avoided by using the dropout layers (10% neurons) in each hidden layer. The dropout layer randomly drops 10% of neurons in each iteration. The structure of the optimal deep learning model and MSE during the training process are illustrated in Figure 5 and Figure 6.

### 2.4. Correlation Matrix Analysis

**Figure 7:**
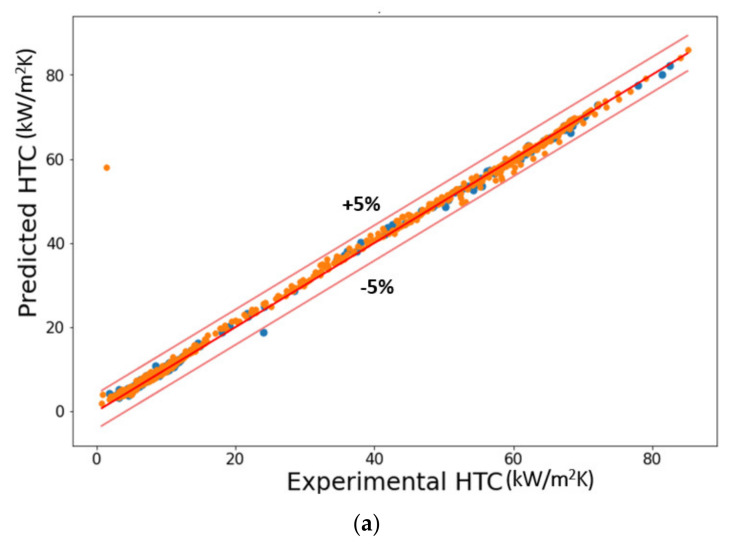

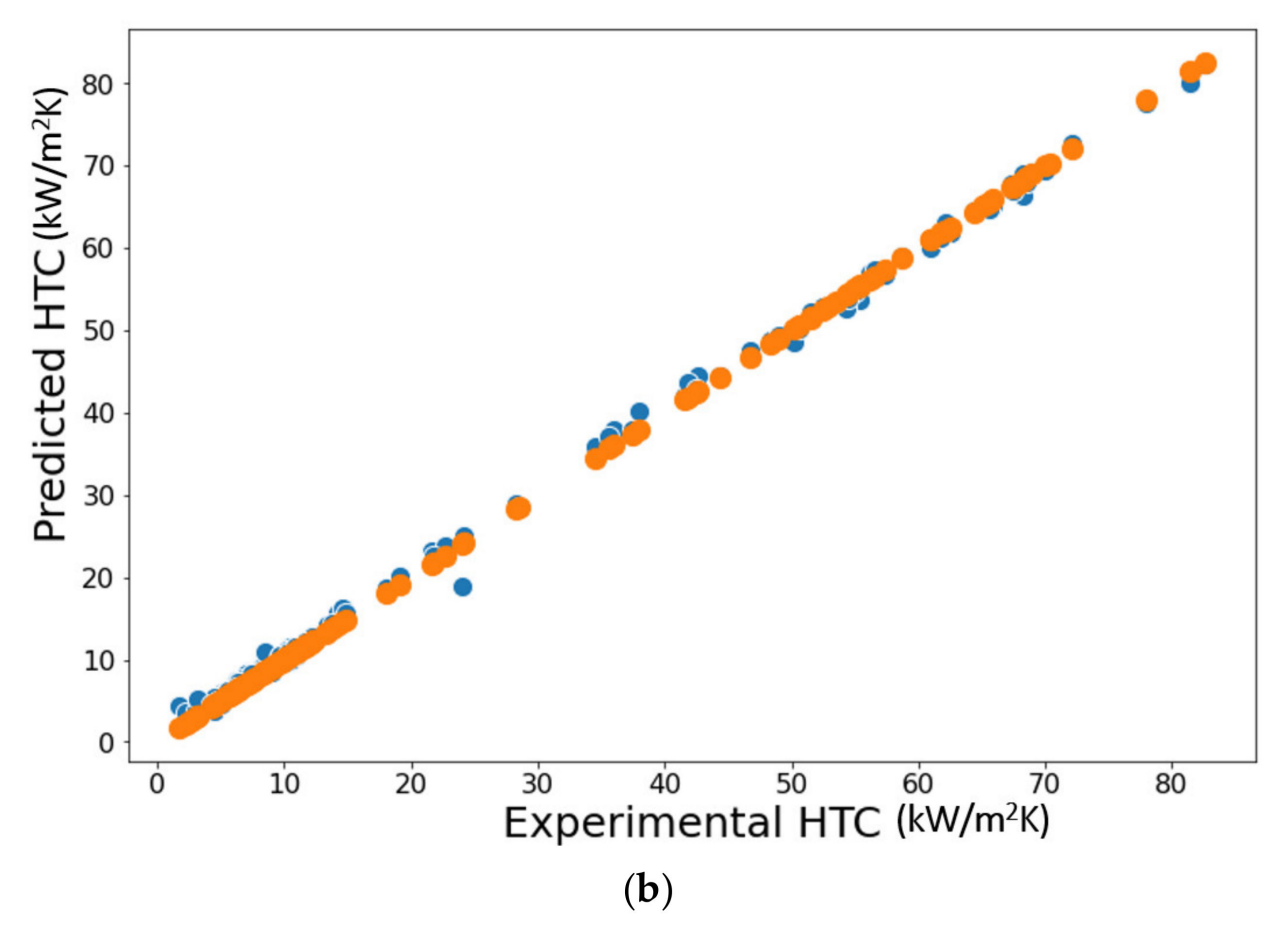
Predictions of the proposed model, (**a**) training and testing datasets and (**b**) testing dataset.

**Figure 8:**
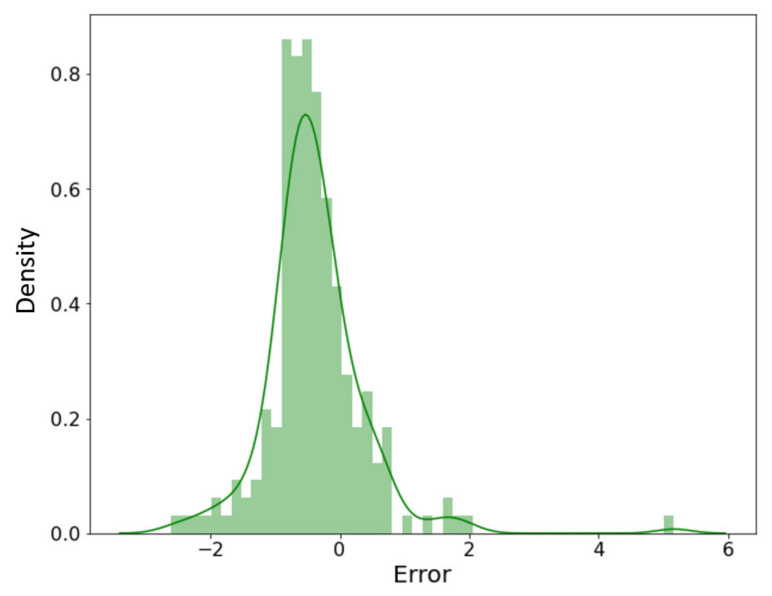
Error density analysis.

**Figure 9:**
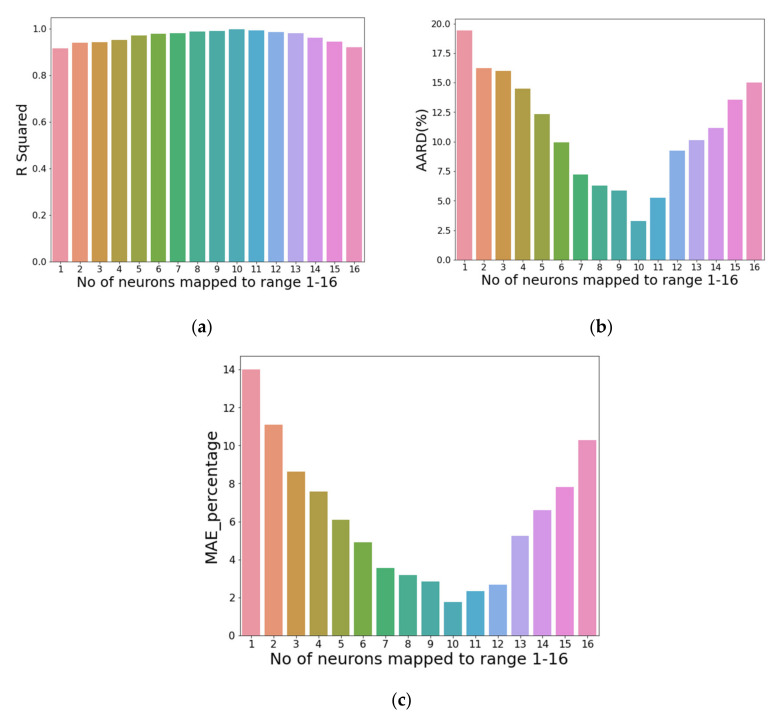
Variation in (**a**) R^2^, (**b**) AARD%, and (**c**) MAE% with the different number of dense neurons.

Figure 7 shows the prediction of the developed model. Error density in Figure 8 shows that the best performance occurred at mapping no. 10. Variation in R^2^, AARD%, and MAE% with the different neurons in the hidden layers can be seen in Figure 9. Table 2 shows the mapping (1–16) of the hidden neurons. Additionally, the mapping no. 10 (45, 45, 0.1) has the optimal value in terms of R^2^ = 0.9988, AARD% = 3.272, and MAE% = 1.77. Figure 9 also shows that by increasing the number of neurons in the hidden layers after a certain limit (mapping no.10), the model’s predictive accuracy tends to decline, and the values of different loss functions such as AARD%, MAE%, and R^2^ are increased. This is due to overfitting caused by the greater number of neurons. In order to avoid overfitting, dropout layers with the different numbers of neurons were used.

## 3. Results and Discussion

**Figure 10:**
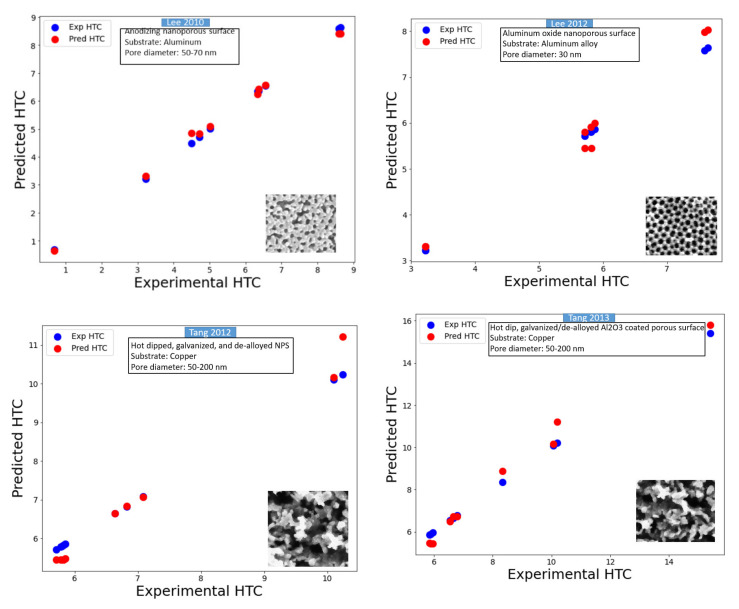

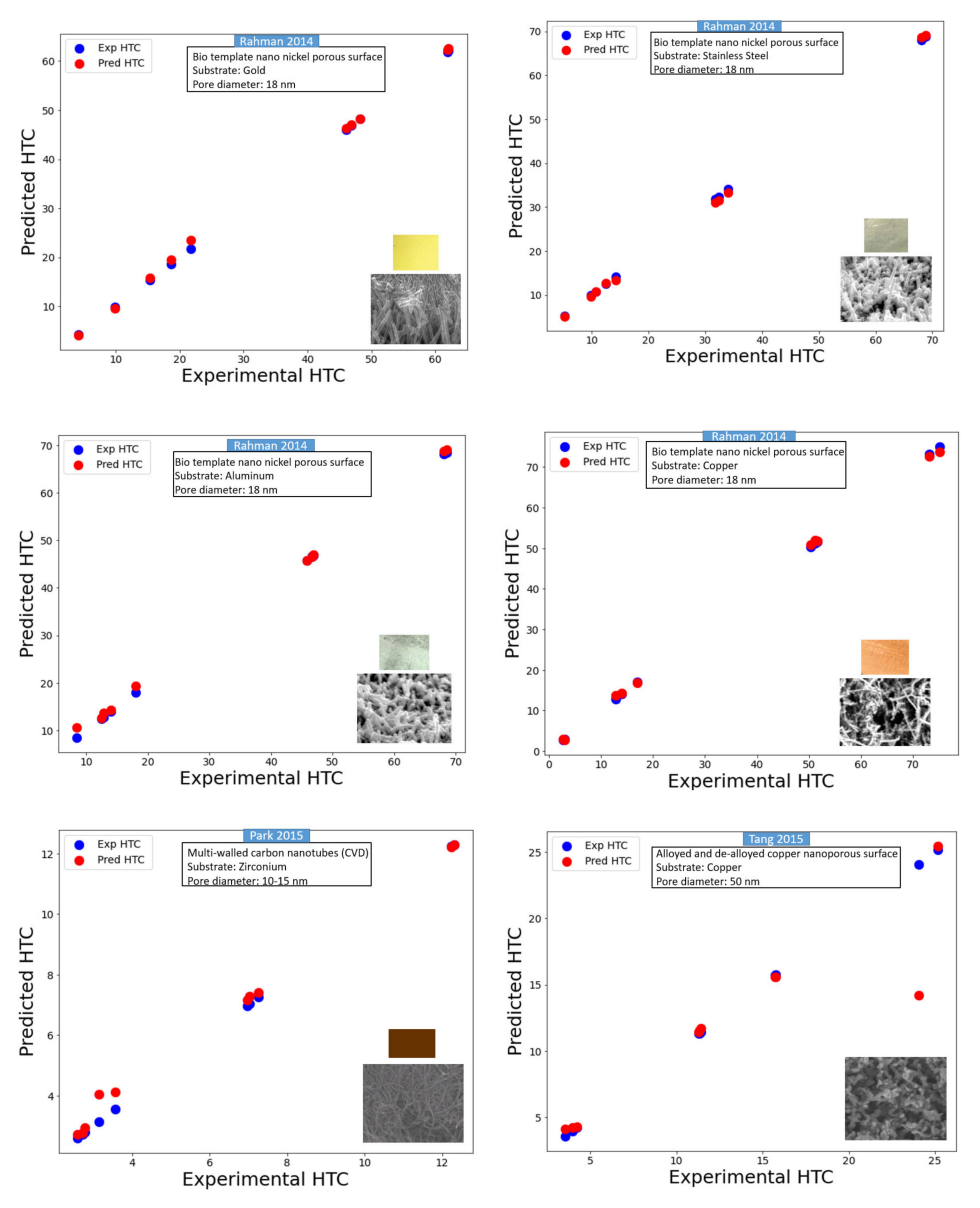

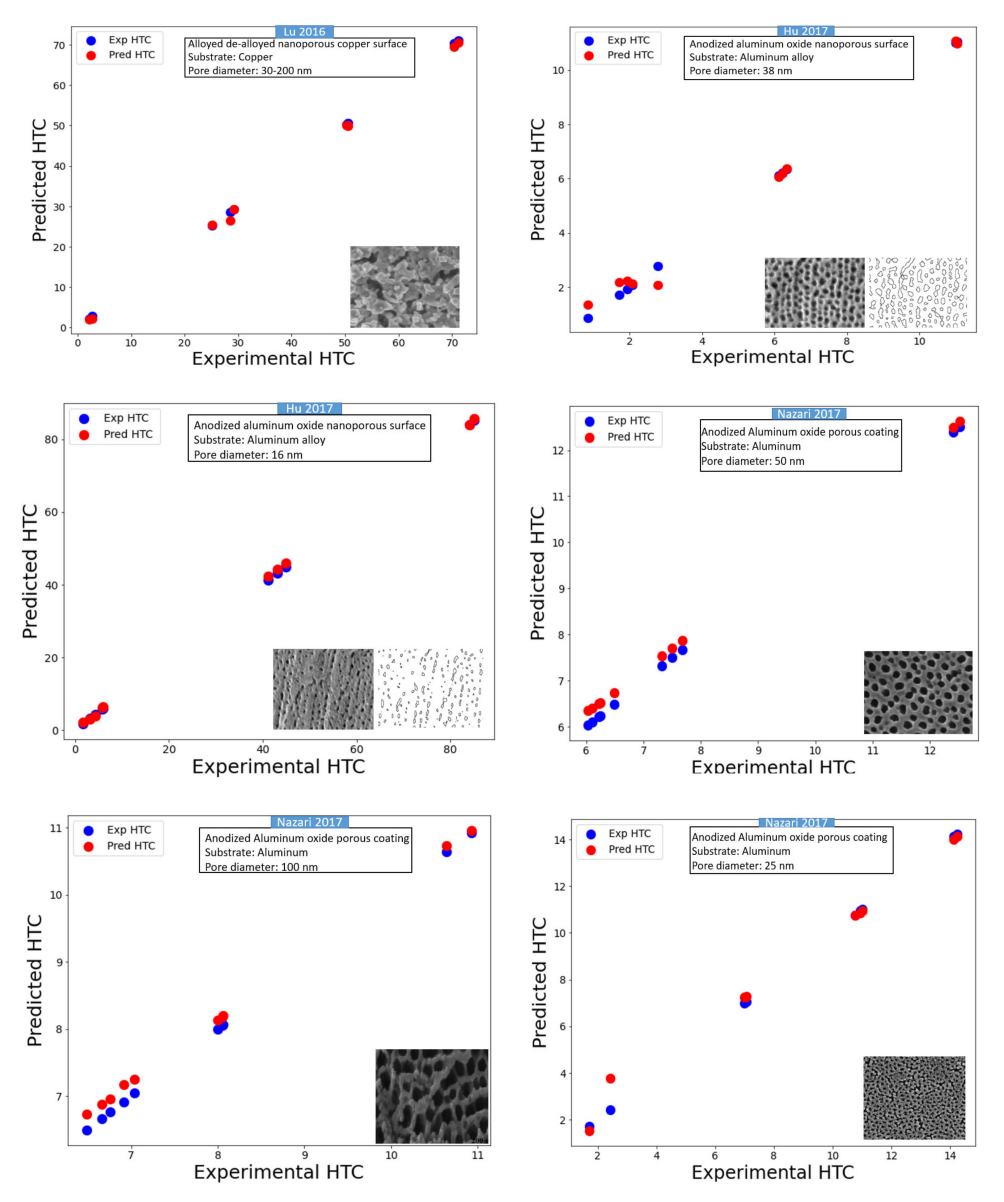

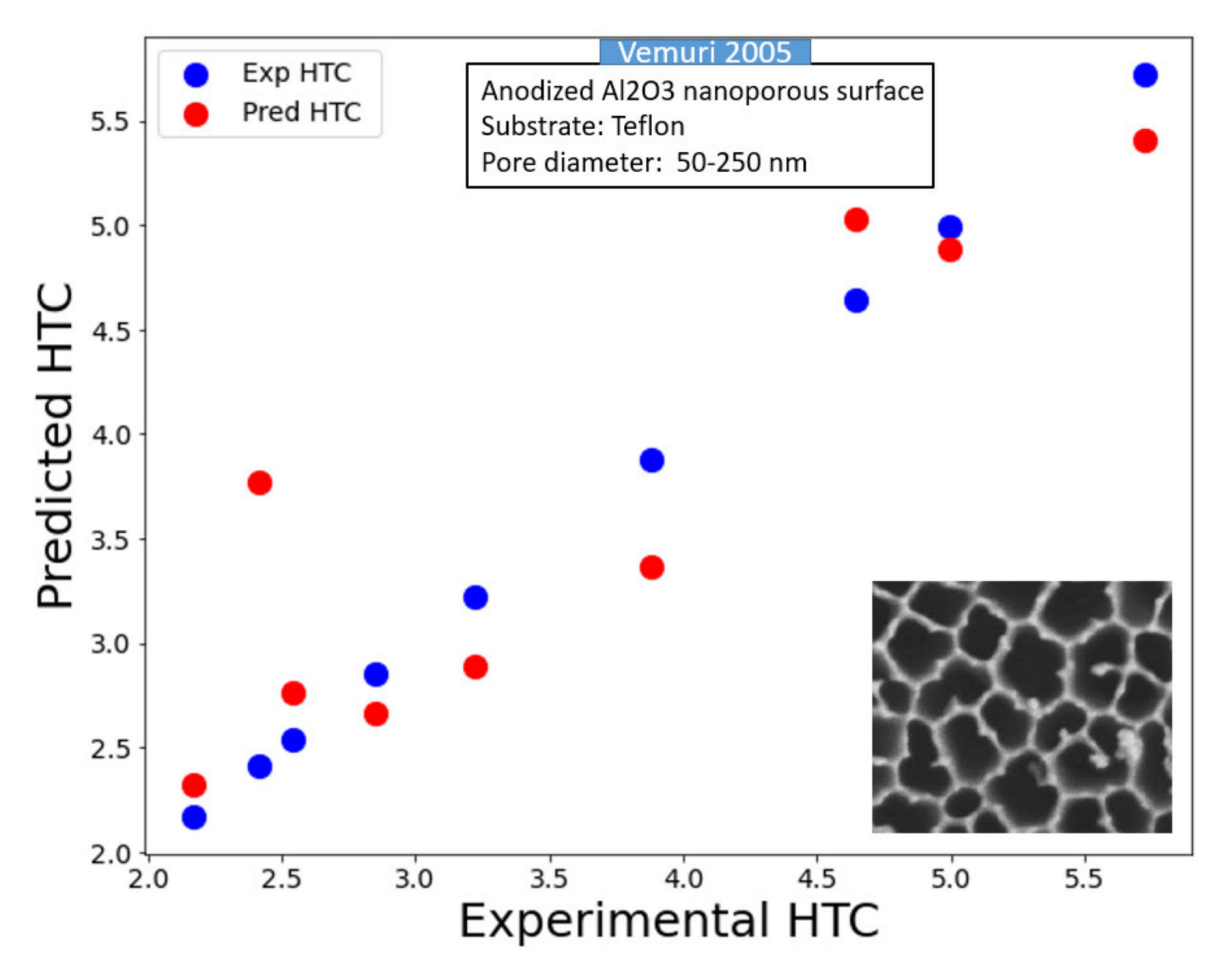
Assessment of various nanoporous coated surfaces available in the literature (note that HTC was taken in kW/m^2^.K).

Figure 10 presents the assessment results of the various nanoporous coated surfaces. Notice that the developed model can predict the saturated pool boiling heat transfer coefficient for various types of nanoporous coated surfaces (Al_2_O_3_ nanoporous coatings, alumina coatings, nickel nanostructures, copper nano-coatings, ZnO nanostructures, Cu_2_O, CNT-Cu composite coatings, Cu-CNT-Al coatings, CNT and graphene coatings, and pHEMA coatings) with different surface shapes (such as flower-like, fractal-like, and dendritic) produced by various fabrication methods (anodization and pyrolysis, cold spraying, electrostatic spraying and sintering, chemical vapor deposition, hot-dip galvanizing/de-alloying, microreactor-assisted nanoparticle deposition, electroplating, and using biological templates) on a variety of substrates (such as copper, aluminum, zirconium, teflon, brass, silicon, stainless steel, gold, and NiCr for both plain and tubular). It is worth mentioning that the prediction was extended for several working fluids with completely different thermophysical properties. In addition, the prediction was still valid for a wide range of heat flux and morphological parameters (such as pore diameter). Individual predictions of the different nanostructures subject to different working fluids can be seen in Figure 10.

**Figure 11:**
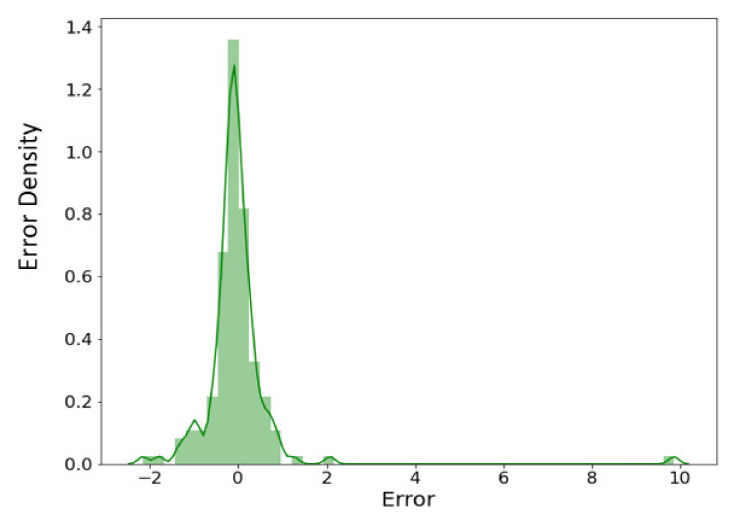
Error density analysis of the predicted results.

The predictions shown in Figure 10 are compared with the nanostructures in the literature. Very highly accurate prediction (an accuracy of almost 99%) of numerous nanoporous coated surfaces for different kinds of working fluids is evident from the error density analysis provided in Figure 11. These predictions can be extended to the varying pressures, surface inclinations, and other boiling conditions (such as subcooling) by introducing the corresponding parameters as the inputs.

As is known, the boiling heat transfer coefficient (BHTC or HTC) mainly relies on the surface morphology, liquid thermophysical properties, and pool boiling testing conditions. The prediction accuracy of any model or correlation relies on how well these parameters are defined or included in the architecture of the model. The existing correlations, due to their theoretical footings, have a very limited range of applications. However, the proposed method includes the most influential parameters into its architecture; thus, the prediction accuracy of this method is substantially higher.

### 3.1. Comparison with the Existing Correlations

**Figure 12:**
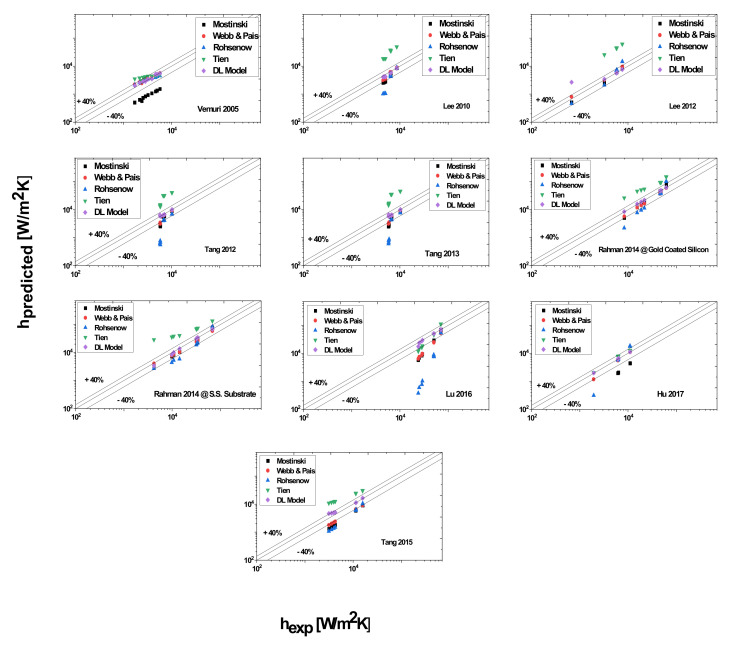
Predictions with correlations available in literature and DL Model.

Data predicted by Rohsenow Rohsenow [40], Tien [41], Mostinski [42], Webb and Paris [43], and the DL model are presented in Figure 1. It can be seen that for most of the cases, the Tien [41] hydrodynamic model over-predicted the data. However, these deviations can be significantly reduced by varying the suggested empirical constant of 61.3. In addition, this model was developed by considering the bubble dynamics on a plain surface, while bubbles on the enhanced geometries behave differently. Apart from that, this model also ignores the vapor properties, and this could be one of the sources of errors. Contrary to the Tien model [41], the Mostinski model underestimated most of the data, which is obvious because the correlation only includes reduced and critical pressure and heat flux, while the thermophysical properties of liquid and surface properties are ignored. As surface roughness is directly related to the amount of vapor trapped, and has a nominal effect on the heat transfer, it seems that these correlations [41,42] can also be improved by incorporating the surface roughness parameter as well. Similarly, most of the predictions by the Webb and Paris correlation [43] based on enhanced tubes also varied within ± 40%. As depicted in Figure 12, the Rohsenow correlation [40] also under-predicted the data in most of the cases. Most of the under-predictions were at low wall superheat. It appears that low wall superheat is responsible for the underestimation. In addition, the different thermophysical properties can be considered as one of the reasons for the discrepancy in prediction capability of the above correlations. Additionally, most of these correlations do not include the vapor properties so this could also be the cause of errors.

Overall, the large deviation of ±40% also indicates the inefficiency of these correlations, which could be because of the selection of inappropriate (or insufficient) functional dependence of the heat transfer coefficient. Moreover, in all cases, we can see that the deep learning model outperformed all empirical correlations and that it predicted accurately. The reason is that this model includes various parameters affecting heat transfer performance and it was trained on a large dataset obtained through the boiling of various working fluids on diverse geometries under varying operational conditions.

### 3.2. Sensitivity Analysis (PBHTC Prediction)

**Figure 13:**
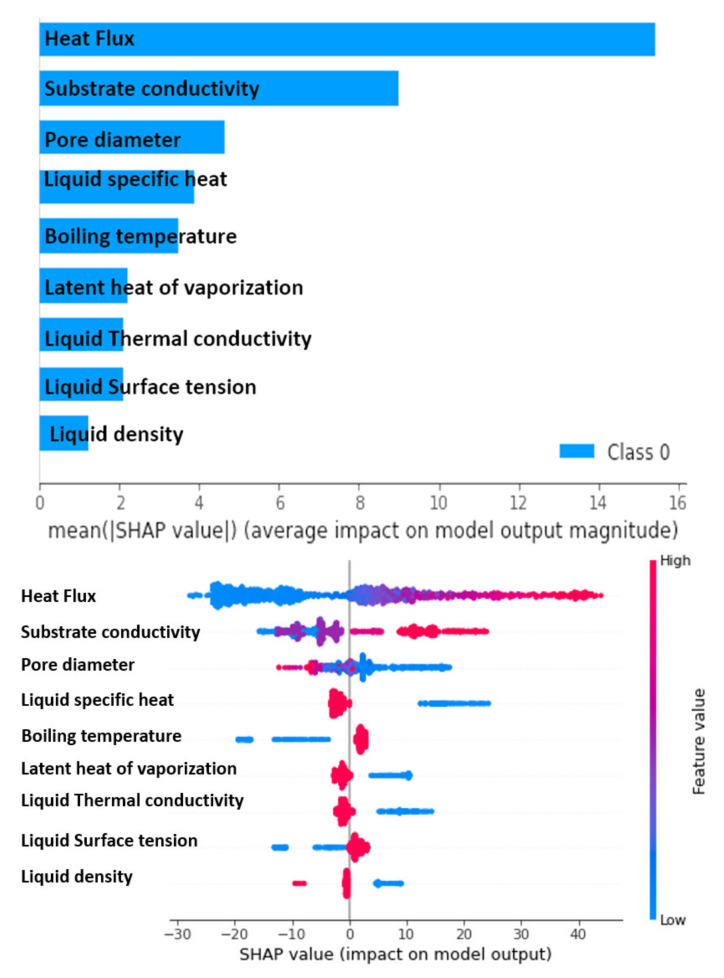
Sensitivity analysis.

From Figure 13, it is apparent that heat flux, the thermal conductivity of the substrate material, and pore diameter are the most influential parameters for the prediction of the HTC of the nanoporous coated surfaces. It is to be noted that heat flux and the thermal conductivity of the substrate are positively correlated with the HTC, while the pore diameter is negatively correlated with the HTC. This is in line with the well-known shreds of evidence that the HTC is augmented by increasing the heat flux, especially in the nucleate boiling regime. Contrarily, the smaller pore diameter is beneficial in increasing the bubble departure frequency. A similar trend of these parameters can be observed in the experimental findings (see Figure 1).

It is stated that the PBHTC of any coated nanoporous surface depends on the heat flux, pore diameter, and thermal conductivity of the substrate material. Therefore, these parameters can be modelled in order to have a quick and accurate estimation of the HTC. Moreover, this investigation can be extended to any working fluid by introducing its thermophysical features in the model.

**Figure 14:**
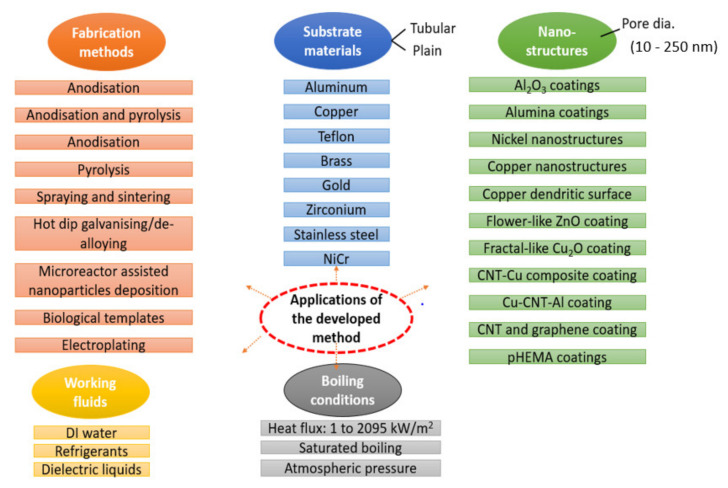
Applications of the proposed method.

The proposed method’s applications with respect to the substrate and coating materials, type of nanostructures, testing conditions, and working fluids can be seen in Figure 14. The proposed deep learning method is based on neural networks consisting of an input layer, hidden layers, and an output layer. The input layer includes input neurons for the input features, such as surface morphological features, liquid thermophysical properties, and pool boiling conditions. The hidden or dense neurons learn from the existing data to relate the inputs and output. This is how a data-driven model learns and predicts the considered output parameter. The main advantage of the proposed method is its applicability for a range of nanostructures, substrate materials, working fluids, and testing conditions. This is how the limitations of the existing correlations can be overcome. As discussed, the proposed method includes all the most impactful parameters into the architecture of the model, which is a major problem in the case of the existing correlations. In addition, the proposed method is simple, quick, and robust. The only limitation is the availability of the data for training.

## 4. Conclusions and Future Outlook

In this study, a deep learning model (DL) was developed that offers a highly accurate estimation of the nucleate boiling heat transfer coefficient of nanoporous coated surfaces subjected to several working fluids under saturated pool boiling conditions. The structure of the developed model includes some of the most influential surface features (such as pore diameter and thermal conductivity of the substrate), as well as the liquid thermophysical properties (such as boiling point, surface tension, specific heat, latent heat of vaporization, etc.) and the pool boiling testing parameters, such as heat flux. In addition to its simplicity and the high accuracy of the prediction, the model is also applicable in a wide range of the investigated parameters (heat flux = 0.3 to 2095 kW/m^2^). The deployment of the liquid thermophysical features makes it suitable for any working fluids. An accuracy of R^2^ = 0.998 is attainable for predicting various kinds of nanostructures. The introduction of the thermal conductivity of the substrate makes it useful for predicting the HTC of nanoporous coatings on any substrate material. The simple, fast, and accurate predictability of the model makes it suitable for boiling applications on nanoporous coated surfaces.

The proposed method can be extended for a wide range of working fluids and nanoscale surfaces for different testing conditions (saturated and subcooled, surface inclinations, the effect of pressure). Moreover, the proposed method highlights the importance of some of the most influential parameters in the pool boiling enhancement of nanoscale surfaces.

## Figures and Tables

**Table 1:** Range of the considered parameters [14,16,18,19,20,21,23,24,25,26,27,28,29,30,31,32,33,34,35,36,37,38,39].

Feature	Range
Heat flux (kW/m^2^)	0.285–2095
Boiling point (°C)	−195.8–100
Specific heat (J/kg⋅K)	1040–4182
Liquid thermal conductivity (W/m⋅K)	0.026–0.580
Pore dia. (nm)	10–250
Substrate thermal conductivity (W/m⋅K)	0.25–401
Heat transfer coefficient (kW/m^2^⋅K)	0.675–85.14
Liquid density (kg/m^3^)	808.4–1680
Surface tension (mN/m)	10–72
Heat of vaporization (kJ/kg)	88–2257.7

**Table 2:** Mapping of the dense neurons.

Sr.#	No. of Neurons in Two Hidden Layers with Dropout	No. of Neurons Mapped to Range 1–16
0	(28, 14)	1
1	(30, 15)	2
2	(40, 20)	3
3	(45, 22)	4
4	(45, 30)	5
5	(45, 40)	6
6	(45, 45, 0.0)	7
7	(45, 45, 0.5)	8
8	(45, 45, 0.3)	9
9	(45, 45, 0.1)	10
10	(46, 45)	11
11	(47, 45)	12
12	(48, 45)	13
13	(50, 45)	14
14	(55, 45)	15
15	(55, 50)	16

## Data Availability

Data are contained within the article.

## References

[1] Sajjad U., Sadeghianjahromi A., Ali H.M., Wang C.-C. (2021). Enhanced pool boiling of dielectric and highly wetting liquids-A review on surface engineering. Appl. Therm. Eng..

[2] Abbas N., Hussain M., Zahra N., Ahmad H., Muhammad S., Mehdi Z., Sajjad U., Amer M. (2020). Optimization of Cr Seed Layer Effect for Surface Roughness of As-Deposited Silver Film using Electron Beam Deposition Method. J. Chem. Soc. Pak..

[3] Abbas N., Shad M.R., Hussain M., Mehdi S.M.Z., Sajjad U. (2019). Fabrication and characterization of silver thin films using physical vapor deposition, and the investigation of annealing effects on their structures. Mater. Res. Express.

[4] Tran N., Sajjad U., Lin R., Wang C.-C. (2020). Effects of surface inclination and type of surface roughness on the nucleate boiling heat transfer performance of HFE-7200 dielectric fluid. Int. J. Heat Mass Transf..

[5] Sajjad U., Abbas A., Sadeghianjahromi A., Abbas N., Liaw J.-S., Wang C.-C. (2021). Enhancing corrosion resistance of Al 5050 alloy based on surface roughness and its fabrication methods; an experimental investigation. J. Mater. Res. Technol..

[6] Sajjad U., Wang C.-C. (2020). Nucleate pool boiling of high flux sintered coated porous surfaces with dielectric liquid, HFE-7200. J. Enhanc. Heat Transf..

[7] Sajjad U., Sadeghianjahromi A., Wang C.-C. (2021). Enhancing boiling heat transfer for electronics cooling by embedding an array of microgrooves into sandblasted surfaces. Heat Transf. Res..

[8] Sajjad U., Sadeghianjahromi A., Ali H.M., Wang C.-C. (2020). Enhanced pool boiling of dielectric and highly wetting liquids-a review on enhancement mechanisms. Int. Commun. Heat Mass Transf..

[9] Tang Y., Tang B., Li Q., Qing J., Lu L., Chen K. (2013). Pool-boiling enhancement by novel metallic nanoporous surface. Exp. Therm. Fluid Sci..

[10] Zhang B.J., Kim K.J. (2014). Nucleate pool boiling heat transfer augmentation on hydrophobic self-assembly mono-layered alumina nano-porous surfaces. Int. J. Heat Mass Transf..

[11] Zhang B.J., Kim K.J., Yoon H. (2012). Enhanced heat transfer performance of alumina sponge-like nano-porous structures through surface wettability control in nucleate pool boiling. Int. J. Heat Mass Transf..

[12] Zhang B.J., Park J., Kim K.J. (2013). Augmented boiling heat transfer on the wetting-modified three dimensionally-interconnected alumina nano porous surfaces in aqueous polymeric surfactants. Int. J. Heat Mass Transf..

[13] Vemuri S., Kim K.J. (2005). Pool boiling of saturated FC-72 on nano-porous surface. Int. Commun. Heat Mass Transf..

[14] Rahman M.M., Ölçeroğlu E., McCarthy M. (2014). Scalable Nanomanufacturing of Virus-templated Coatings for Enhanced Boiling. Adv. Mater. Interfaces.

[15] Zheng X., Park C.W. (2015). Experimental study of the sintered multi-walled carbon nanotube/copper microstructures for boiling heat transfer. Appl. Therm. Eng..

[16] Lu L., Fu T., Tang Y., Tang T., Tang B., Wan Z. (2016). A novel in-situ nanostructure forming route and its application in pool-boiling enhancement. Exp. Therm. Fluid Sci..

[17] Tang Y., Tang B., Qing J., Li Q., Lu L. (2012). Nanoporous metallic surface: Facile fabrication and enhancement of boiling heat transfer. Appl. Surf. Sci..

[18] Gao J., Lu L.-S., Sun J.-W., Liu X.-K., Tang B. (2017). Enhanced boiling performance of a nanoporous copper surface by electrodeposition and heat treatment. Heat Mass Transf..

[19] Pialago E.J.T., Kwon O.K., Jin J.S., Park C.W. (2016). Nucleate pool boiling of R134a on cold sprayed Cu–CNT–SiC and Cu–CNT–AlN composite coatings. Appl. Therm. Eng..

[20] Hendricks T.J., Krishnan S., Choi C., Chang C.-H., Paul B. (2010). Enhancement of pool-boiling heat transfer using nanostructured surfaces on aluminum and copper. Int. J. Heat Mass Transf..

[21] Hassanpour M., Vaferi B., Masoumi M.E. (2018). Estimation of pool boiling heat transfer coefficient of alumina water-based nanofluids by various artificial intelligence (AI) approaches. Appl. Therm. Eng..

[22] Sajjad U., Hussain I., Hamid K., Bhat S.A., Ali H.M., Wang C.-C. (2021). A deep learning method for estimating the boiling heat transfer coefficient of porous surfaces. J. Therm. Anal. Calorim..

[23] Zarei M., Ansari H., Keshavarz P., Zerafat M. (2020). Prediction of pool boiling heat transfer coefficient for various nano-refrigerants utilizing artificial neural networks. J. Therm. Anal. Calorim..

[24] Khalili Sadaghiani A., Reza Motezakker A., Volkan Özpınar A., Özaydın İnce G., Koşar A. (2017). Pool boiling heat transfer characteristics of inclined pHEMA-coated surfaces. J. Heat Transf..

[25] Lee C.Y., Bhuiya M.M.H., Kim K.J. (2010). Pool boiling heat transfer with nano-porous surface. Int. J. Heat Mass Transf..

[26] Fazel S.A.A. (2017). A genetic algorithm-based optimization model for pool boiling heat transfer on horizontal rod heaters at isolated bubble regime. Heat Mass Transf..

[27] Liu Y., Dinh N., Sato Y., Niceno B. (2018). Data-driven modeling for boiling heat transfer: Using deep neural networks and high-fidelity simulation results. Appl. Therm. Eng..

[28] Može M., Zupančič M., Golobič I. (2020). Investigation of the scatter in reported pool boiling CHF measurements including analysis of heat flux and measurement uncertainty evaluation methodology. Appl. Therm. Eng..

[29] Arya M., Khandekar S., Pratap D., Ramakrishna S.A. (2016). Pool boiling of water on nano-structured micro wires at sub-atmospheric conditions. Heat Mass Transf..

[30] Hu H., Xu C., Zhao Y., Ziegler K.J., Chung J. (2017). Boiling and quenching heat transfer advancement by nanoscale surface modification. Sci. Rep..

[31] Jo H.S., An S., Park H.G., Kim M.-W., Al-Deyab S.S., James S.C., Choi J., Yoon S.S. (2017). Enhancement of critical heat flux and superheat through controlled wettability of cuprous-oxide fractal-like nanotextured surfaces in pool boiling. Int. J. Heat Mass Transf..

[32] Jones P.R., Elliott A.R., Patankar N.A. (2016). Sustaining superheated liquid within hydrophilic surface texture. Langmuir.

[33] June Zhang B., Kim K.J. (2012). Effect of liquid uptake on critical heat flux utilizing a three dimensional, interconnected alumina nano porous surfaces. Appl. Phys. Lett..

[34] Kalaiselvam S., Gugan M., Kuraloviyan E., Meganathan R., Priyan A.N., Swaminathan M. (2009). Experimental investigation of anodized/spray pyrolysed nanoporous structure on heat transfer augmentation. J. Therm. Sci..

[35] Lee C.Y., Zhang B.J., Kim K.J. (2012). Morphological change of plain and nano-porous surfaces during boiling and its effect on nucleate pool boiling heat transfer. Exp. Therm. Fluid Sci..

[36] Nazari A., Saedodin S. (2017). Porous anodic alumina coating for optimisation of pool-boiling performance. Surf. Eng..

[37] Park S.-S., Kim Y.H., Jeon Y.H., Hyun M.T., Kim N.-J. (2015). Effects of spray-deposited oxidized multi-wall carbon nanotubes and graphene on pool-boiling critical heat flux enhancement. J. Ind. Eng. Chem..

[38] Pialago E.J.T., Kwon O.K., Park C.W. (2013). Nucleate boiling heat transfer of R134a on cold sprayed CNT–Cu composite coatings. Appl. Therm. Eng..

[39] Tang B., Zhou R., Lu L., Zhou G. (2015). Augmented boiling heat transfer on a copper nanoporous surface and the stability of nano-porosity in a hydrothermal environment. Int. J. Heat Mass Transf..

[40] Rohsenow W.M. (1951). A Method of Correlating Heat Transfer Data for Surface Boiling of Liquids.

[41] Tien C. (1962). A hydrodynamic model for nucleate pool boiling. Int. J. Heat Mass Transf..

[42] Mostinski I. (1963). Application of the rule of corresponding states for calculation of heat transfer and critical heat flux. Teploenergetika.

[43] Webb R.L., Pais C. (1992). Nucleate pool boiling data for five refrigerants on plain, integral-fin and enhanced tube geometries. Int. J. Heat Mass Transf..

